# Orthodontic camouflage of skeletal Class III malocclusion with miniplate: a case report

**DOI:** 10.1590/2177-6709.21.4.089-098.oar

**Published:** 2016

**Authors:** Marcel Marchiori Farret, Milton M. Benitez Farret, Alessandro Marchiori Farret

**Affiliations:** 1 Professor, Centro de Estudos Odontológicos Meridional (CEOM), Graduate Program in Orthodontics, Passo Fundo/RS, Brazil; and Fundação para Reabilitação das Deformidades Crânio-faciais (FUNDEF), Lajeado/RS, Brazil.; 2 Professor, Universidade Federal de Santa Maria (UFSM), Santa Maria/RS, Brazil.; 3 Private practice, Santa Maria/RS, Brazil.

**Keywords:** Angle Class III malocclusion, Orthodontic anchorage procedures, Orthodontic appliance design.

## Abstract

**Introduction:**

Skeletal Class III malocclusion is often referred for orthodontic treatment combined with orthognathic surgery. However, with the aid of miniplates, some moderate discrepancies become feasible to be treated without surgery.

**Objective:**

To report the case of a 24-year-old man with severe skeletal Angle Class III malocclusion with anterior crossbite and a consequent concave facial profile.

**Methods:**

The patient refused to undergo orthognathic surgery; therefore, orthodontic camouflage treatment with the aid of miniplates placed on the mandibular arch was proposed.

**Results:**

After 18 months of treatment, a Class I molar and canine relationship was achieved, while anterior crossbite was corrected by retraction of mandibular teeth. The consequent decrease in lower lip fullness and increased exposure of maxillary incisors at smiling resulted in a remarkable improvement of patient's facial profile, in addition to an esthetically pleasing smile, respectively. One year later, follow-up revealed good stability of results.

## RESUMO

**Introdução:**

a má oclusão de Classe III esquelética é frequentemente tratada com associação entre Ortodontia e Cirurgia Ortognática. No entanto, com o auxílio das miniplacas, discrepâncias moderadas tornaram-se passíveis de serem tratadas sem a necessidade de cirurgia ortognática.

**Objetivo:**

relatar o caso de um paciente de 24 anos, com má oclusão de Classe de III Angle e Classe III esquelética, mordida cruzada anterior e consequente perfil facial côncavo.

**Métodos:**

o paciente recusou-se a realizar a cirurgia ortognática, assim, foi proposta a compensação da má oclusão com o auxílio de miniplacas na arcada inferior.

**Resultados:**

após 18 meses de tratamento, foram obtidas relações de molares e caninos em Classe I, e a mordida cruzada anterior foi corrigida, por meio da distalização de todos os dentes inferiores. A consequente redução na projeção do lábio inferior, associada à maior exposição dos incisivos superiores no sorriso, resultou em um perfil facial consideravelmente mais agradável e um sorriso esteticamente melhor, respectivamente. A análise um ano após o tratamento revelou boa estabilidade dos resultados obtidos.

## INTRODUCTION

Skeletal Class III malocclusion is one of the biggest challenges faced by orthodontists.^1^ ^,^ ^2^ If patients consent to orthognathic surgery, subsequent mechanical orthodontic treatment becomes simple with superior functional and esthetic results.^3^ ^,^ ^4^ ^,^ ^5^ However, several patients refuse surgery. In such situations, orthodontic camouflage treatment may be an alternative, particularly if discrepancy is slight or moderate.^3^ ^,^ ^4^ ^,^ ^6^

The introduction of skeletal anchorage has increased the number of patients with skeletal problems who can be treated by mechanical orthodontic treatment only, thereby avoiding the need for complementary orthognathic surgery.^2^ ^,^ ^7^ Mini-implants are preferred for patients with slight discrepancies because of less invasive insertion and removal procedures.^8^ ^,^ ^9^ However, in patients with moderate skeletal and dental discrepancies, miniplates are the treatment of choice to improve anchorage and eliminate the possibility of contact between implant screws and tooth roots during tooth movement, as it can occur with mini-implants.^2^ ^,^ ^8^ ^,^ ^10^ ^,^ ^11^

In the present study, we report the case of a 24-year-old man with severe skeletal Angle Class III malocclusion who was treated by orthodontic camouflage treatment with miniplate anchorage. 

## CASE REPORT

### Diagnosis and etiology

**Figure 1:**
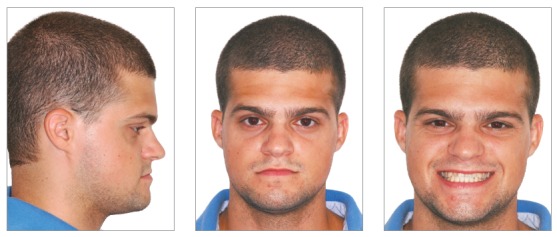
Pretreatment facial photographs.

**Figure 2:**
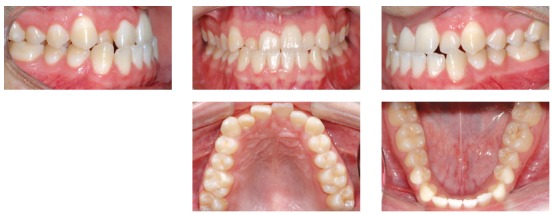
Pretreatment intraoral photographs.

**Figure 3:**
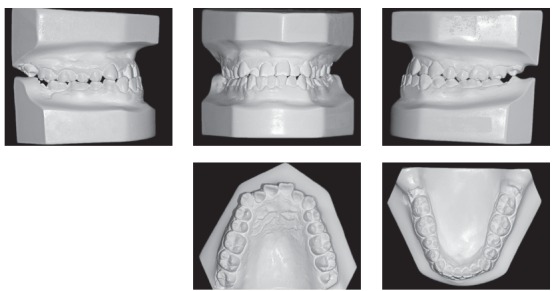
Pretreatment dental casts.

**Figure 4:**
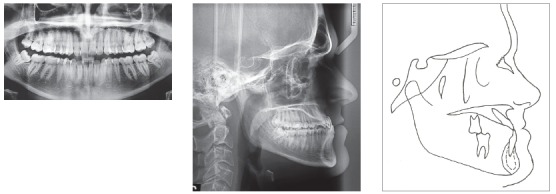
Pretreatment radiographs and pretreatment cephalogram.

A 24-year-old man presented for orthodontic treatment with the chief complaint of an unesthetic smile. Undesired appearance was caused by protrusion of anterior teeth and decreased visibility of maxillary anterior teeth at smiling. Extraoral examination revealed a concave facial profile (Fig 1). Clinical manipulation in centric relation demonstrated that there was no mandibular anterior deviation during bite closing. Intraoral examination and analysis of dental casts revealed Angle Class III malocclusion, Class III canine relationship, anterior crossbite, and maxillary incisor crowding, with a negative discrepancy of 4 mm (Figs 2 and 3). Furthermore, Bolton analysis revealed 1-mm excess for maxillary posterior teeth and 2-mm excess for mandibular anterior teeth. Cephalometric analysis revealed skeletal Class III (ANB = −5°) malocclusion, a hypodivergent facial pattern (SN-GoGn = 20°, FMA = 9° and Y-axis = 44°), severe maxillary incisor proclination, and uprighted mandibular incisors (Fig 4). 

### Treatment objectives

The primary treatment objectives for this patient were: (1) establish a Class I molar and canine relationship; (2) correct anterior crossbite and achieve adequate overjet and overbite; (3) eliminate maxillary incisor crowding; and (4) improve facial esthetics by straightening the facial profile and increasing maxillary incisor exposure at smiling. 

### Treatment alternatives

The first treatment option for this patient was orthognathic surgery for maxillary advancement, which would certainly improve facial esthetics and simplify subsequent mechanical orthodontic treatment; however, the patient refused to undergo surgery. The second option was mechanical orthodontic treatment with Class III elastics and a sliding jig on the mandibular arch. This would require prolonged use of elastics with extremely good patient compliance and could result in some undesirable effects, such as counterclockwise occlusal plane rotation, with less maxillary incisor and greater mandibular incisor exposure. The third option was the use of mini-implants as anchorage unit; which was disregarded because the required tooth movement was extensive and the mini-implant would require removal and relocation at some point during treatment. Eventually, camouflage orthodontic treatment with miniplate anchorage was proposed and the patient agreed with this option. In this planning, treatment would be started with alignment and leveling of lower and upper arches, except for maxillary incisors, thus avoiding further proclination. After alignment and leveling of the upper arch, stripping was considered from second molar to first premolar on each side, so as to gain space for incisors alignment. On the lower arch, after alignment and leveling, miniplates would be inserted on each side of the posterior mandible, so to be used as the anchorage unit to retract all mandibular teeth. During anterior crossbite correction, a posterior bite plate was also planned to be used, so as to avoid interferences between maxillary and mandibular incisors. 

### Treatment progress

**Figure 5:**
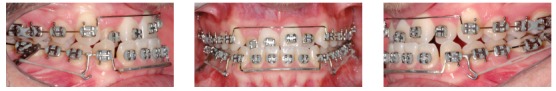
Intraoral photographs at the beginning of mandibular dentition distalization.

Treatment was initiated by bonding 0.022 × 0.028-in Edgewise standard brackets followed by alignment and leveling of both arches with 0.014-in and 0.016-in Nickel-Titanium archwires and 0.016-in, 0.018-in, and 0.020-in stainless steel archwires. The archwires were not inserted for incisors to avoid proclination and premature contact between maxillary and mandibular incisors. Stripping from the mesial surface of the maxillary second molar to the mesial surface of the maxillary first premolar was performed on both sides, followed by distalization of all maxillary posterior teeth. Mandibular posterior teeth were aligned and leveled up to a 0.020-in stainless steel archwire, and at this point in treatment, miniplates were placed on the external oblique ridge. Subsequently, a 0.019 × 0.025-in stainless steel archwire was set with hooks between the canine and first premolar on both sides and connected to the miniplates by means of elastomeric chains, thus resulting in a load of 400 g/f on each side (Fig 5). 

**Figure 6:**
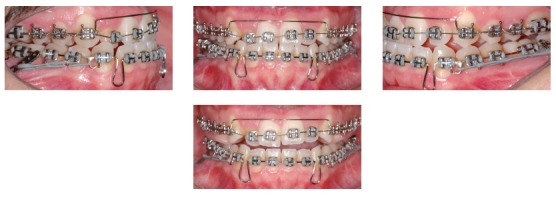
Intraoral photographs when maxillary and mandibular incisors were included on the mechanics to correct anterior crossbite.

**Figure 7:**
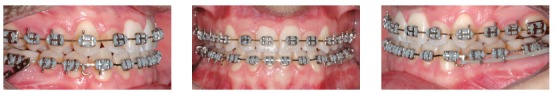
Intraoral photographs after anterior crossbite correction.

After two months, an improved anteroposterior (AP) relationship was achieved, and maxillary and mandibular incisors were included in treatment (Fig 6). An overlayed 0.012-in nickel-titanium archwire was placed in the maxillary arch to align the incisors with slight proclination, while a 0.019 × 0.025-in stainless steel archwire was set with bull loops and placed in the mandibular arch to retract the incisors. This archwire was activated on the miniplates, and another elastomeric chain was connected to the mandibular first premolar on each side to maintain mandibular dentition retraction. To facilitate anterior crossbite correction, a removable posterior bite plate was used for two months. After 14 months, anterior crossbite was completely corrected and a Class I molar and canine relationship was achieved. At this point, upper and lower 0.019 × 0.025-in stainless steel archwires were placed to achieve the appropriate torque, with elastomeric chains connected only on the left miniplate, so as to correct slight midline deviation (Fig 7). 

### Treatment results 

**Figure 8:**
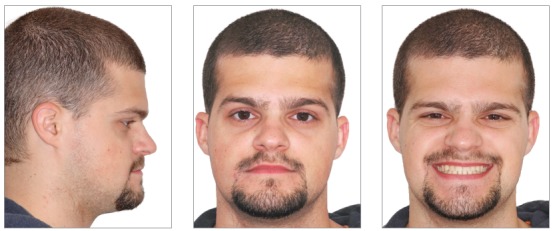
Post-treatment facial photographs.

**Figure 9:**
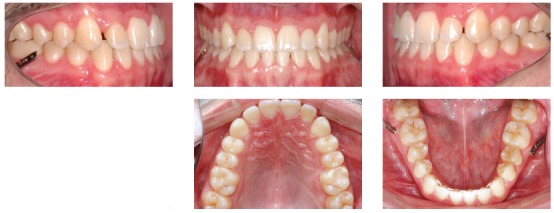
Post-treatment intraoral photographs.

**Figure 10:**
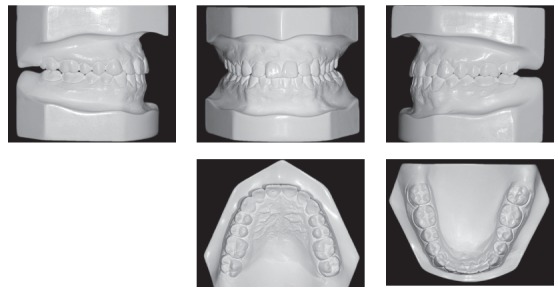
Post-treatment dental casts.

**Figure 11:**
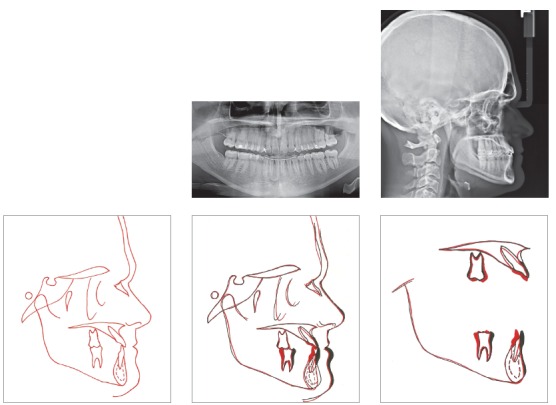
Post-treatment radiographs, post-treatment cephalogram, total superimposition and partial superimpositions.

**Figure 12:**
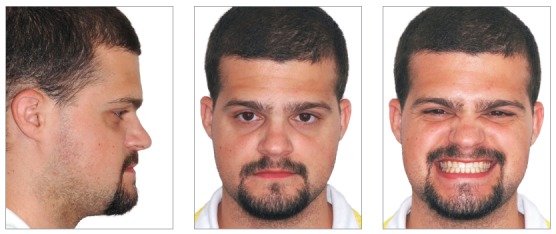
1-year post treatment facial photographs.

**Figure 13:**
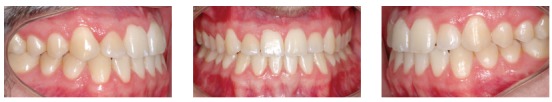
1-year post-treatment intraoral photographs.

Patient's treatment was complete after 18 months. His facial profile remarkably improved with an esthetically pleasing smile (Fig 8). Intraoral examination and dental casts analysis revealed a Class I molar and canine relationship on both sides, with excellent intercuspation (Figs 9 and 10). Due to anterior Bolton discrepancy, spaces were kept unchanged between maxillary lateral incisors and canines, which would be filled with composite resin. Anterior crossbite was successfully corrected and adequate overjet and overbite were achieved. Panoramic radiograph showed good parallelism among tooth roots, and cephalometric analysis with superimpositions revealed that maxillary incisors remained nearly at the same position, with mandibular molar uprighting and distalization and high mandibular incisors retraction, with a consequent decrease in lower lip fullness (Fig 11). Fortunately, one year after treatment follow-up showed that the occlusion remained stable, with molar and canine in Class I relationship and good intercuspation (Figs 12 and 13).

**Table 1:** Cephalometric measurements.

Measurements	Norms (SD)	Initial	Post-treatment
SNA	82° (3)	86	85
SNB	80° (3)	91	90
ANB	2° (2)	-5	-5
Facial convexity (NA.APog)	0° (2)	-13	-14
Facial angle (PoOr.NPog)	87° (3)	103	102
Y-axis	59° (6)	44	45
SN.GoGn	32° (3)	20	21
1-NA (°)	22°	44	41
1-NA (mm)	5 mm	6	8
1-NB (°)	25°	13	5
1-NB (mm)	5 mm	3	0
Inter-incisal angle	131° (5)	126	139
Ul-S line	0 mm (2)	-5	-4
Ll-S line	0 mm (2)	-1	-3
IMPA	90° (4)	85	74
FMA	25° (3)	9	11
FMIA	65° (4)	86	95

## DISCUSSION

The present article reported the case of a 24-year-old man with severe skeletal Angle Class III malocclusion. The patient was treated by orthodontic camouflage treatment with miniplate anchorage. In the last few years, only slight skeletal discrepancies in adult patients were usually treated without orthognathic surgery.^9^ Treatment options included the use of Class III elastics alone or in combination with a sliding jig or even headgears, stripping, and tooth extraction.^3^ ^,^ ^6^ ^,^ ^10^ Unfortunately, all these options were associated with complications, such as counterclockwise rotation of the occlusal plane,^2^ ^,^ ^4^ ^,^ ^12^ ^,^ ^13^ patient's noncompliance with elastics or headgears,^14^ ^,^ ^15^ patient's refusal to undergo extraction, and the creation of Bolton discrepancy in cases of stripping. The advent of skeletal anchorage increased the reliability of results because it does not require patient compliance and it is associated with minimal or no side effects. In this context, miniplates represent the best option for simultaneous multiple tooth movement because of the increased stability generated by multiple screws instead of a single screw as with mini-implants. Conventionally, miniplates are inserted at two sites in Class III patients: on the external oblique ridge with the active end positioned at the mesial or distal surface of the first molar or on the lower border of the mandible with the active end positioned at the mesial surface of the first molar.^10^ For the presented case, the surgeon faced some difficulty during the procedure and had to fix right and left miniplates with their active ends around the mesial and distal surfaces of the first molar, respectively, with no mechanical issues thereafter.

In patients with moderate skeletal Class III malocclusion, one question must always be addressed by orthodontists: is it possible to camouflage this malocclusion? There are several parameters influencing this decision. First, the extent of compromise on facial esthetics, and whether compromise is a big concern for the patient must be judged.^4^ ^,^ ^5^ ^,^ ^13^ In the present study, the patient was not hugely concerned about his facial esthetics, and profile concavity was moderate. Certainly, if the patient's chief complaint was facial esthetics, orthognathic surgery, and not camouflage treatment alone, would be necessary. The second parameter is the anteroposterior position and angulation of maxillary and mandibular incisors. In patients with an edge-to-edge anterior bite or a slight anterior crossbite, correction can be achieved after judging the extent of maxillary incisor proclination and mandibular incisor retroclination. Our patient showed severe maxillary incisor proclination; however, mandibular incisors were not retroclined, thereby facilitating orthodontic camouflage by means of incisor retraction. The third parameter is thickness of mandibular symphysis, which should be adequate to allow extensive incisor retraction.^3^ Fortunately, in our patient, the anteroposterior dimension of the symphysis was adequate. Finally, the last parameter is the degree of anteroposterior discrepancy. Even if facial esthetics is acceptable, the symphysis is thick enough, and mandibular incisors are slightly proclined, camouflage is not possible if anteroposterior discrepancy is too severe. Considering that anteroposterior discrepancy was moderate in the patient reported herein, orthodontic camouflage was selected.

One major concern for orthodontists is stability of camouflage treatment after mandibular incisor retraction in patients with Class III malocclusion.^5^ ^,^ ^14^ Considering that the entire arch is retracted by 4-5 mm, the tongue has less space after treatment, thus resulting in extreme tongue pressure on mandibular incisors and consequent relapse with premature contact between incisors and abnormal spacing between mandibular teeth.^10^ ^,^ ^16^ Some alternatives to improve stability in such cases include achieving an ideal overjet, overbite, and intercuspation;^2^ ^,^ ^5^ maintenance of mandibular posterior teeth in an upright position after distalization because distal tipping tends cause them to return to their original position according to their root apices;^10^ ^,^ ^14^ ^,^ ^15^ using a 3 × 3 bonded mandibular retainer for an undetermined period of time;^13^ myofunctional therapy to eliminate tongue interposition during swallowing and rest; and to position the tip of the tongue at the incisive papilla during swallowing and in the posterior region of the oral cavity at rest.^17^

Superimpositions at follow-up revealed excessive remodeling of the symphysis because of mandibular incisor retraction. Incisors centered on the symphysis at the beginning of treatment maintained the centers at the end of treatment, thus avoiding gingival recession in the long-term and improving stability.^5^ In the case presented herein, analysis one year after treatment revealed excellent stability of results. The patient will remain under post-treatment follow-up once a year. 

## CONCLUSION

In the case reported herein, miniplates proved to be reliable as anchorage unit for mandibular dentition distalization and camouflage of skeletal Class III, thus avoiding orthognathic surgery. 
